# Clinicopathological characteristics and assessment of risk factors for incidentally discovered prostatic cancer in patients undergoing radical cystectomy with urothelial carcinoma of the bladder

**DOI:** 10.3389/fonc.2026.1704699

**Published:** 2026-04-22

**Authors:** Ying Qiu, Nai-Chun Zhang, Xiao Tan, Ning Jin, Da Chen, Jing Zhao

**Affiliations:** 1 Department of Pathology, Linyi People′s Hospital, Linyi, Shandong, China; 2 Department of Infectious Disease, Linyi People’s Hospital, Linyi, Shandong, China

**Keywords:** assessment of risk factors, bladder urothelial carcinoma, HER2, incidental prostatic adenocarcinoma, radical cystectomy

## Abstract

**Background:**

This retrospective study analyzed data from patients undergoing radical cystoprostatectomy (RCP) for bladder urothelial carcinoma. We analyzed their features with regard to prevalence, clinicopathological characteristics and evaluated the risk of incidental prostatic adenocarcinoma (IPCa).

**Methods:**

Clinical data and pathological features of 237 patients who underwent standard RCP for bladder urothelial carcinoma were included in this study. Correlations between bladder tumor size, number of tumors, histological grade, pathological stage, patient age, and incidental prostate cancer were examined. Human epidermal growth factor receptor 2 (HER2) expression was evaluated using immunohistochemistry (IHC), and its association with clinicopathological features was analyzed.

**Results:**

Among the 237 patients, 69 (29.1%) had bladder urothelial carcinoma complicated by IPCa. Age was identified as a risk factor for IPCa (OR = 1.04, 95% CI: 1.00-1.07, *P* = 0.038), corresponding to a 4% increase in risk per additional year of age. In patients with bladder urothelial carcinoma, there was a statistically significant difference in the maximum tumor diameter among the three HER2 expression groups (0/1+, 2+ and 3+, *P* = 0.041). Specifically, the median maximum tumor diameter in the HER2 2+ group was 3.20 cm, which was lower than that in the other two groups (both 4.00 cm). Additionally, the distribution of histological subtypes differed significantly among the three HER2 groups (*P* = 0.024), with the proportion of conventional urothelial carcinoma being the highest in the HER2 0/1+ group (86.6%).

**Conclusions:**

IPCa is not uncommon in patients undergoing radical cystectomy. It is of great significance to evaluate the risk factors for informing surgical strategy and postoperative management. Our analysis further demonstrates that HER2 expression status correlates significantly with tumor size and histology in urothelial carcinoma. Specifically, tumors with HER2 2+ expression exhibited a smaller median diameter, while the classic histological subtype was predominantly observed in HER2 0/1+ tumors. These associations indicate that differential HER2 expression may delineate distinct pathological phenotypes of bladder urothelial carcinoma.

## 1 Introduction

Bladder urothelial carcinoma is a common malignancy of the urinary system in elderly men. RCP, involving resection of the bladder, prostate, seminal vesicles, and regional lymph nodes, serves as the standard surgical modality for treating muscle-invasive bladder cancer in male patients (1). However, this procedure frequently leads to complications such as urinary incontinence and erectile dysfunction, which significantly impaires patients’ quality of life. To improve patients’ quality of life while ensuring the curative effect of bladder cancer treatment, prostate-sparing cystectomy (PSC), which retains all or part of the prostate, has been developed (2, 3). A critical clinical challenge arises from the frequent detection of IPCa in RCP specimens. IPCa is a malignancy that is clinically occult, undetectable by palpation or imaging, and incidentally diagnosed histopathologically in surgical specimens from non-prostate cancer procedures. Currently, preoperative prediction of concomitant IPCa in bladder urothelial carcinoma patients remain unreliable. This study aims to investigate the clinicopathological characteristics and identify risk factors associated with IPCa in RCP specimens, thereby providing evidence to guide clinical decision-making.

## 2 Materials and methods

### 2.1 Inclusion criteria

Clinical and pathological data of 237 patients who underwent RCP for bladder urothelial carcinoma in the Department of Urology of our hospital from January 2017 to January 2026 were collected. All patients underwent CT scan or MRI examination before surgery to assess the stage of bladder cancer. The inclusion criteria were as follows: (1) histologically confirmed bladder urothelial carcinoma, (2) complete clinicopathological data available.

### 2.2 Methods

#### 2.2.1 Tissue processing and staining

All tissue specimens were fixed in 10% neutral buffered formalin, routinely processed through graded alcohols, embedded in paraffin, and sectioned at 4 μm thickness. Sections were stained with hematoxylin and eosin (H&E) for routine histological examination, with immunohistochemical (IHC) staining performed as needed for diagnostic confirmation.

#### 2.2.2 Immunohistochemistry

IHC staining was performed using an automated staining system (Ventana Medical Systems, USA) with the following primary antibodies: CK7, CK20, CK5/6, ki-67 (MIB-1 clone), GATA-3, S100P, P504S (AMACR), p63, and HER2. All primary antibodies were obtained from Zhongshan Golden Bridge Biotechnology (Beijing, China). The staining procedures were strictly performed according to the manufacturers’ protocols.

### 2.3 Treatment and pathological examination

At our urology center, patients with non-muscle-invasive bladder cancer (NMIBC) are primarily treated with transurethral resection of bladder tumor (TURBT), while those with muscle-invasive bladder cancer (MIBC) routinely receive neoadjuvant chemotherapy for three months prior to radical cystectomy. Both the bladder cancer specimens and the prostate tissue within radical cystectomy specimens were processed and sampled in a standardized manner, in accordance with surgical pathology guidelines and relevant recommendations from the International Society of Urological Pathology (ISUP). For the prostate tissue specifically, the three-dimensional parameters of the prostate were measured, and its external surfaces (left, right, anterior and posterior) were inked for margin assessment. The bilateral ureteral margin, urethral margin and prostatic apical margin were preferentially sampled and submitted for embedding. For the apical margin, tissue sections were obtained at 4–5 mm from the apex perpendicular to the urethra, followed by sequential sectioning at 2–3 mm intervals parallel to the urethra.

The remaining prostatic tissue was transversely sectioned at 3–4 mm thickness, divided into four parts (left anterior, left posterior, right anterior and right posterior) in a cross-shaped pattern along the urethra for sampling. Routine preparation yielded 50–80 sections per prostate specimen. If seminal vesicles were attached, both were longitudinally bisected for complete sampling.

All pathological slides were independently reviewed by two pathologists to evaluate the pathological characteristics of bladder urothelial carcinoma with IPCa. Both bladder cancer and prostate cancer were diagnosed and classified according to the 2022 WHO Classification of Tumours of the Urinary and Male Genital Systems.

### 2.4 Evaluation of HER2 expression

Positive HER2 signals are localized on the cell membrane. The IHC evaluation criteria refer to the 2021 “Chinese expert consensus on clinical pathology of human epidermal growth factor receptor 2 detection in urothelial carcinoma”. A four-point scoring system was applied: 0: No staining or <10% of invasive cancer cells show incomplete, weak membranous staining.1+: ≥10% of invasive cancer cells show incomplete, weak membranous staining. 2+: ≥10% of invasive cancer cells show weak to moderate intensity complete membranous staining, or <10% of invasive cancer cells show strong and complete membranous staining. 3+: ≥10% of invasive cancer cells show strong, complete, and uniform membranous staining. Based on the scores, patients were divided into three groups: HER2 0/1+ group, HER2 2+ group, and HER2 3+ group. Subsequently, the association between HER2 protein expression level and various clinicopathological characteristics was analyzed.

### 2.5 Statistical analysis

Statistical analyses were performed using SPSS version 25.0. Normality of continuous variables was assessed using the Shapiro–Wilk test. Variables with a normal distribution (*P* > 0.05) were further tested for homogeneity of variance. Those meeting both normality and homogeneity of variance assumptions were expressed as mean ± standard deviation (x̄ ± s), and comparisons between groups were conducted using the independent-samples t-test or analysis of variance (ANOVA). Non-normally distributed variables were presented as median with interquartile range [M (P25–P75)], and comparisons were performed using the Wilcoxon rank-sum test or Kruskal–Wallis test. Categorical variables were summarized as frequency and percentage [n (%)], and between-group differences were assessed using the χ² test or Fisher’s exact test. Potential risk factors for IPCa were identified using univariate and multivariate logistic regression analyses; variables with *P* ≤ 0.10 in the univariate analysis were entered into the multivariate model. Survival curves were generated using the Kaplan–Meier method. A significance level of α= 0.05 was used, and *P* < 0.05 was considered statistically significant.

## 3 Results

### 3.1 Clinical characteristics

A total of 237 patients with high-grade urothelial carcinoma were included in this study (**Table 1**). A pooled analysis of preoperative biopsy specimens from all patients showed that the vast majority of cases were pathologically diagnosed as high-grade urothelial carcinoma, with the invasive subtype being predominant. A small number of cases were initially reported as low-grade urothelial carcinoma with focal high-grade components on biopsy, however, these cases were confirmed as high-grade carcinoma in the final radical cystoprostatectomy specimens. Regarding treatment, 10 patients initially underwent transurethral resection of bladder tumor. All of these patients experienced multiple recurrences postoperatively and eventually underwent RCP. The age of patients ranged from 38 to 100 years, with a median age of 69 years. Among them, 42 cases (17.7%) were ≤60 years old, while 195 cases (82.3%) were >60 years old. Regarding the histological subtypes of bladder cancer, there were 201 cases of classic urothelial carcinoma, 19 cases with squamous differentiation, 2 cases with squamous differentiation and glandular differentiation, 5 cases were of the micropapillary subtype, 7 cases with glandular differentiation, 1 case with micropapillary differentiation and glandular differentiation, 1 case with micropapillary differentiation and trophoblastic differentiation, and 1 case with pseudoglandular and micropapillary differentiation. Additionally, the maximum tumor diameter ranged from 0.8 to 12 cm, with a median of 4.0 cm. Multifocal bladder cancer was observed in 67 cases (28.3%). For pT staging: 15 cases (6.3%) were at pTa, 76 cases (32.1%) at pT1, 69 cases (29.1%) at pT2, 53 cases (22.4%) at pT3, and 24 cases (10.1%) at pT4. The majority of patients underwent standard lymph node dissection. Postoperative pathological evaluation revealed that the total number of lymph nodes dissected per patient ranged from 1 to 29, among which the number of lymph nodes positive for metastatic bladder cancer ranged from 1 to 8. According to the pathological nodal (pN) staging: 142 cases (59.9%) were pN0, 18 cases (7.6%) were pN1, 8 cases (3.4%) were pN2, and 3 cases (1.3%) were pN3. Additionally, 66 cases (27.8%) were classified as pNX. Furthermore, among the entire cohort of 237 patients, concurrent IPCa was identified in 69 cases (29.1%).

**Table 1:** Clinical characteristics of patients with bladder cancer.

Factor	Group without IPCa (n=168)	Group with IPCa (n=69)	Total (n=237)	*P*-value
Age (years), M (P25-P75)	69.0 (62.0 - 73.0)	70.0 (63.0 - 77.0)	69.0 (63.0 - 75.0)	0.140
≤60	31 (18.5)	11 (15.9)	42 (17.7)	0.646
>60	137 (81.5)	58 (84.1)	195 (82.3)	
Maximum tumor diameter (cm), M (P25-P75)	3.5 (2.5 - 5.0)	4.0 (2.7 - 4.5)	4.0 (2.5 - 5.0)	0.771
Number (n, %)				
Single	119 (70.8)	51 (73.9)	170 (71.7)	0.632
Multiple	49 (29.2)	18 (26.1)	67 (28.3)	
Histological subtype (n, %)				
Classic urothelial carcinoma	140 (83.3)	61 (88.4)	201 (84.8)	0.818
Urothelial carcinoma with micropapillary differentiation	4 (2.4)	1 (1.4)	5 (2.1)	
Urothelial carcinoma with squamous differentiation	14 (8.3)	5 (7.2)	19 (8.0)	
Urothelial carcinoma with multidirectional differentiation (trophoblastic differentiation and micropapillary atypia)	1 (0.6)	0	1 (0.4)	
Urothelial carcinoma with pseudoglandular and micropapillary differentiation	1 (0.6)	0	1 (0.4)	
Urothelial carcinoma with squamous differentiation and glandular differentiation	2 (1.2)	0	2 (0.8)	
Urothelial carcinoma with glandular and micropapillary differentiation	0	1 (1.4)	1 (0.4)	
Urothelial carcinoma with glandular differentiation	6 (3.6)	1 (1.4)	7 (3.0)	
pT stage of bladder cancer (n, %)				
Ta	9 (5.4)	6 (8.7)	15 (6.3)	0.417
T1	59 (35.1)	17 (24.6)	76 (32.1)	
T2	48 (28.6)	21 (30.4)	69 (29.1)	
T3	36 (21.4)	17 (24.6)	53 (22.4)	
T4	16 (9.5)	8 (11.6)	24 (10.1)	
pN stage of bladder urothelial carcinoma (n, %)				
N0	102 (60.7)	40 (58.0)	142 (59.9)	0.116
N1	13 (7.7)	5 (7.2)	18 (7.6)	
N2	7 (4.2)	1 (1.4)	8 (3.4)	
N3	0	3 (4.3)	3 (1.3)	
NX	46 (27.4)	20 (29.0)	66 (27.8)	
HER2 results of urothelial carcinoma (n, %)				
0	46 (27.4)	19 (27.5)	65 (27.4)	0.866
1+	43 (25.6)	19 (27.5)	62 (26.2)	
2+	62 (36.9)	20 (29.0)	82 (34.6)	
3+	17 (10.1)	11 (15.9)	28 (11.8)	
PSA (ng/ml), M (P25-P75)	0.67 (0.46 - 1.91)	1.08 (0.64 - 2.31)	0.76 (0.55 - 2.15)	0.203
Muscle-invasive bladder urothelial carcinoma (n, %)				
NMIBC	68 (40.5)	23 (33.3)	91 (38.4)	0.304
MIBC	100 (59.5)	46 (66.7)	146 (61.6)	

### 3.2 Clinicopathological characteristics of bladder urothelial carcinoma patients with IPCa

The ages of 69 patients diagnosed with postoperative pathological combined IPCa ranged from 48 to 100 years old. The maximum diameter of bladder urothelial carcinoma varied between 1.0 cm and 12 cm. There were 51 cases (73.9%) of single focus and 18 cases (26.1%) of multifocal lesions. For pT staging: 6 cases (8.7%) were at pTa, 17 cases (24.6%) at pT1, 21 cases (30.4%) at pT2, 17 cases (24.6%) at pT3 and 8 cases (11.6%) at pT4. For pN staging: 40 cases (58.0%) were at pN0, 5 cases (7.2%) at pN1, 1 cases (1.4%) at pN2, 3 cases (4.3%) at pN3, and 20 cases (29.0%) at pNX. All prostate cancer cases were histologically classified as acinar adenocarcinoma. According to the Gleason score: 49 cases (71.0%) had a Gleason score of 3 + 3 = 6 (prognostic group 1), 17 cases (24.6%) had a Gleason score of 3 + 4 = 7 (prognostic group 2), 2 cases (2.9%) had a Gleason score of 4 + 3 = 7 (prognostic group 3), and 1 case (1.4%) had a Gleason score of 4 + 4 = 8 (prognostic group 4). Among them, 67 cases (97.1%) were confined to the prostate (pT2), 2 cases (2.9%) had extraprostatic invasion (pT3). According to literature reports, clinically significant IPCa was defined as meeting any of the following criteria: presence of Gleason grade ≥4 components, prostate cancer with stage ≥pT3, lymph node metastasis, positive surgical margins, or detection of 3 or more multifocal lesions. According to histopathological criteria, 20 cases in this cohort were identified as clinically significant IPCa. Among the 237 cases in this group, 65 underwent serum PSA testing. The median preoperative serum PSA level was 1.08 ng/ml (range: 0.64-2.31 ng/ml) in patients with IPCa, and 0.67 ng/ml (range: 0.46-1.91 ng/ml) in those without prostate cancer. This showed no statistically significant difference between the two groups (*P* = 0.203). To identify influencing factors for incidental prostate cancer, logistic regression analysis was conducted. Univariate analysis initially identified age and pN stage as candidate variables (**Table 2**), which were subsequently entered into the multivariate model for adjustment. Multivariate logistic regression analysis revealed that age was a risk factor for bladder cancer complicated with IPCa (OR = 1.04, 95%CI: 1.00-1.07, *P* = 0.038), corresponding to a 4% elevated risk of IPCa per 1-year increase in age (**Table 3**).

**Table 2:** Univariate logistic regression analysis.

Factor	OR,95%CI			*P*-value
	OR value	Lower	Upper	
Age	1.03	1.00	1.06	0.065
Maximum tumor diameter	0.99	0.84	1.17	0.883
PSA	1.02	0.79	1.34	0.861
Number				
Multiple vs Single	0.87	0.46	1.63	0.658
pT stage				
T1 vs Ta	0.43	0.13	1.37	0.154
T2 vs Ta	0.65	0.21	2.05	0.459
T3 vs Ta	0.70	0.22	2.28	0.554
T4 vs Ta	0.75	0.20	2.85	0.676
pN stage				
N1 vs N0	1.03	0.35	3.04	0.956
N2 vs N0	0.51	0.08	3.37	0.481
N3 vs N0	17.73	0.57	554.16	0.102
NX vs N0	1.12	0.59	2.11	0.737
HER2				
1+ vs 0	1.07	0.50	2.28	0.863
2+ vs 0	0.78	0.38	1.63	0.511
3+ vs 0	1.57	0.62	3.96	0.342

pT, pathological tumor stage; pN, pathological nodal stage.

**Table 3:** Multivariate logistic regression analysis.

Factor	OR,95%CI			*P*-value
	OR value	Lower	Upper	
Age	1.04	1.00	1.07	0.038
pN stage				
N1 vs N0	1.02	0.34	3.07	0.979
N2 vs N0	0.29	0.03	2.53	0.265
N3 vs N0	>999.99	<0.00	>999.99	0.986
NX vs N0	1.01	0.53	1.95	0.967

### 3.3 Expression of HER2 in bladder urothelial carcinoma

In 237 patients with urothelial carcinoma, statistically significant differences were observed in tumor maximum diameter and histological subtype distribution across HER2 expression subgroups (0/1+, 2+, 3+, *P* = 0.041 and *P* = 0.024, respectively, **Table 4**). The median tumor maximum diameter was significantly smaller in the HER2 2+ subgroup (3.20 cm) than in both the HER2 0/1+ and 3+ subgroups (each 4.00 cm). Additionally, conventional urothelial carcinoma was most prevalent in the HER2 0/1+ subgroup, accounting for 86.6% of cases. No significant differences were observed among the three subgroups for other clinicopathological features, including patient age, tumor multiplicity, and lymph node metastasis status (pN stage) (all *P*>0.05). To further investigate HER2 expression patterns, we conducted subgroup analyses stratifying patients by bladder cancer invasiveness (NMIBC vs. MIBC) and, within each group, by prostate cancer risk level (low, intermediate, and high). Statistical comparisons were performed to assess differences in HER2 expression across these subgroups. The results showed no significant differences in HER2 expression among any of the subgroups analyzed (all *P* > 0.05).

**Table 4:** Correlation analysis between HER2 expression and clinicopathological characteristics.

Factor	0/1+ (n=127)	2+ (n=82)	3+ (n=28)	*P*-value
Age (years), M (P25-P75)	69.0 (62.0 - 74.0)	69.5 (62.0 - 75.0)	69.0 (66.5 - 74.0)	0.653
≤60	23 (18.1)	16 (19.5)	3 (10.7)	0.567
>60	104 (81.9)	66 (80.5)	25 (89.3)	
Maximum tumor diameter (cm), M (P25-P75)	4.0 (2.7 - 5.0)	3.2 (2.5 - 4.3)	4.0 (3.0 - 4.8)	0.041
Number (n, %)				
Single	96 (75.6)	57 (69.5)	17 (60.7)	0.246
Multiple	31 (24.4)	25 (30.5)	11 (39.3)	
Histological subtype (n, %)				
Classic urothelial carcinoma	110 (86.6)	68 (82.9)	23 (82.1)	0.024
Urothelial carcinoma with micropapillary differentiation	0	3 (3.7)	2 (7.1)	
Urothelial carcinoma with squamous differentiation	14 (11.0)	3 (3.7)	2 (7.1)	
Urothelial carcinoma with multidirectional differentiation (trophoblastic differentiation and micropapillary atypia)	0	1 (1.2)	0	
Urothelial carcinoma with pseudoglandular and micropapillary differentiation	0	1 (1.2)	0	
Urothelial carcinoma with squamous differentiation and glandular differentiation	0	2 (2.4)	0	
Urothelial carcinoma with glandular and micropapillary differentiation	1 (0.8)	0	0	
Urothelial carcinoma with glandular differentiation	2 (1.6)	4 (4.9)	1 (3.6)	
pT stage of bladder urothelial carcinoma (n, %)				
Ta	7 (5.5)	7 (8.5)	1 (3.6)	0.597
T1	41 (32.3)	26 (31.7)	9 (32.1)	
T2	40 (31.5)	22 (26.8)	7 (25.0)	
T3	28 (22.0)	20 (24.4)	5 (17.9)	
T4	11 (8.7)	7 (8.5)	6 (21.4)	
pN stage of bladder urothelial carcinoma (n, %)				
N0	73 (57.5)	52 (63.4)	17 (60.7)	0.122
N1	5 (3.9)	10 (12.2)	3 (10.7)	
N2	4 (3.1)	4 (4.9)	0	
N3	3 (2.4)	0	0	
NX	42 (33.1)	16 (19.5)	8 (28.6)	
Lymph node metastasis of bladder urothelial carcinoma (n, %)				
Metastasis	12 (9.4)	14 (17.1)	3 (10.7)	0.499
No metastasis	73 (57.5)	52 (63.4)	17 (60.7)	
Muscle-invasive bladder urothelial carcinoma (n, %)				
NMIBC	48 (37.8)	33 (40.2)	10 (35.7)	0.895
MIBC	79 (62.2)	49 (59.8)	18 (64.3)	

HER2, human epidermal growth factor receptor 2.

### 3.4 Imaging features of bladder urothelial carcinoma patients with IPCa

All 237 patients underwent preoperative CT scan or MRI for bladder cancer staging. Among them, 31 patients received prostate MRI examinations. Retrospective analysis revealed that these 31 patients all had a prostate Prostate Imaging – Reporting and Data System (PI-RADS) score of 1 or 2. A total of 69 patients in this study were ultimately diagnosed with incidental prostate cancer. Preoperative imaging examinations in these IPCa patients generally showed only prostatic enlargement and/or calcification, with no identifiable nodules or other suspicious findings suggestive of prostate cancer.

### 3.5 Follow-up status and overall survival outcomes

**Figure 1:**
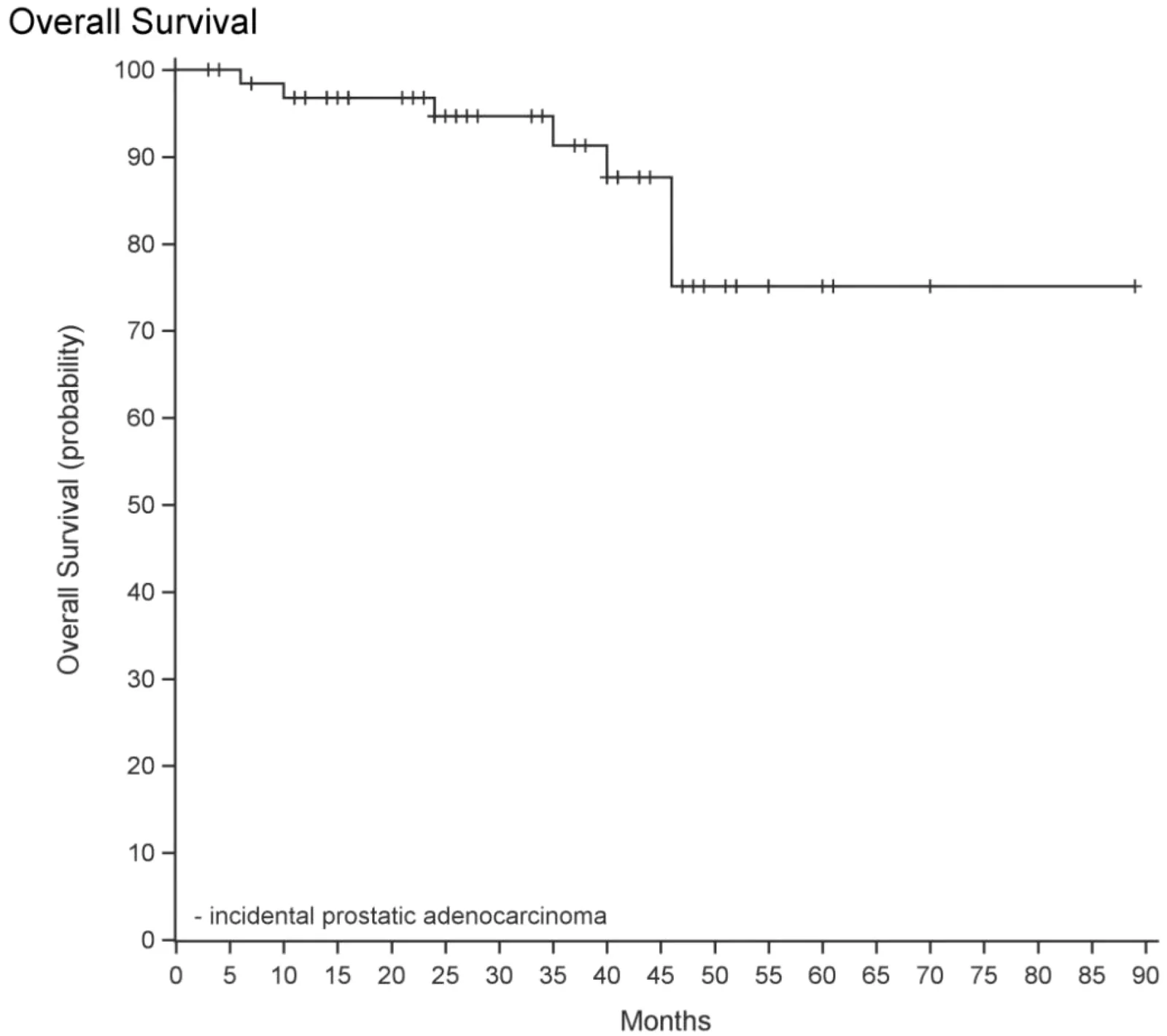
Kaplan–Meier survival curves of bladder cancer patients with IPCa.

A total of 231 patients were successfully followed up, with a follow-up duration ranging from 1 to 108 months. During the entire follow-up period, 23 patients died. Among them, 4 patients died due to advanced age and cardiovascular/cerebrovascular diseases, 18 patients died from recurrence and distant metastasis of bladder cancer, and 1 patient died from liver metastasis of gastrointestinal adenocarcinoma. Among the 69 patients with bladder cancer complicated by IPCa, 65 were followed up, and 4 patients were lost to follow-up, with a median follow-up duration of 33 months (95% CI: 26–40 months). Among them, 7 patients (10.8%) died, of whom 3 succumbed to advanced age and underlying cardiovascular diseases, and 4 died from multiple organ failure resulting from the progression of bladder cancer. The remaining patients are currently in good survival condition. Overall survival analysis of 65 patients with IPCa was performed (**Figure 1**). Survival analysis showed that the 1, 3 and 5-year overall survival rates of the patients were 96.8%, 91.3% and 75.1% respectively. Limited by the sample size for long-term follow-up, more subjects and longer follow up duration are still needed in the future to further clarify the long term survival benefits.

## 4 Discussion

The concurrent presence of bladder cancer and IPCa in RCP specimens represents a clinically relevant phenomenon. Given the unique pathological context of this dual-cancer cohort, the suitability of PSC and its potential implications for IPCa residual or recurrence warrant careful evaluation, as the present study lacks comparative data on different surgical techniques. Consequently, further investigation is warranted to elucidate the clinicopathological features, identify associated risk factors, and establish evidence-based criteria for selecting appropriate candidates for PSC in this dual-cancer cohort.

Pathological analysis of RCP specimens from 237 male bladder cancer patients in this study identified IPCa in 69 cases (29.1%). This incidence is closely aligned with relevant prior reports (4, 5). Supporting this, Harman Maxim Bruins et al. (6)conducted a comprehensive analysis of 1,476 RCP specimens, finding concurrent prostate cancer and urothelial carcinoma involving the prostate in 37.9% and 21.1% of cases, respectively. Similarly, Marco Moschini et al. (7)reported prostate cancer in 35.7% of patients undergoing RCP, with 14.1% exhibiting clinically significant disease. The variation in reported rates of prostate involvement among studies likely reflects differences in the thoroughness of histopathological sampling and evaluation protocols for RCP specimens.

This study confirms that advanced age is a significant risk factor for IPCa in bladder urothelial carcinoma patients, aligning with established literature. We observed a clear age-dependent increase in IPCa incidence among our cohort. Consistent with prior literature (8), we defined clinically significant IPCa by meeting any of the following criteria: presence of Gleason grade ≥4compo-nents, pathological stage≥pT3, lymph node metastasis, positive surgical margins, or≥3 multifocal lesions. Based on these histopathological criteria, 20 cases in this series were classified as clinically significant, accounting for 29% of all IPCa cases.

Currently, there is no established consensus on the clinical management or prognostic significance of IPCa identified in RCP specimens. In support of a conservative management approach, Vignesh T. Packiam et al. (9) reported an IPCa incidence of 20.1% (329/1640) in RCP samples, with the majority of cases classified as clinically insignificant. Long-term follow-up data from their cohort indicated that prostate cancer-specific mortality was exceedingly rare. Corroborating these findings, studies by Simon Jønck and Kaelberer et al. (10, 11) demonstrated that IPCa did not impact overall mortality in RCP patients, irrespective of Gleason score, surgical margin status, or pathological T stage. Crucially, genomic research by Jing et al. (12)demonstrated that bladder cancer and IPCa lesions exhibit unique genomic abnormalities, which indicates the existence of distinct mutational processes in the tumorigenesis of bladder and prostate. Consequently, the authors recommend managing concurrent bladder cancer and IPCa as two independent disease entities. In this study, among the 237 RCP specimens, 69 (29.1%) cases were complicated with IPCa, including 20 cases of clinically significant IPCa. All remaining patients underwent close postoperative monitoring and follow-up. 65 patients with IPCa were followed up for a median duration of 33 months (95%CI: 26–40 months). During the follow-up period, a total of 7 patients died, of whom 3 succumbed to advanced age and underlying cardiovascular diseases, and 4 died from multiple organ failure resulting from the progression of bladder cancer. Four were lost to follow-up, and the rest are currently in good condition. Survival analysis of 65 patients with IPCa revealed a relatively favorable overall prognosis, providing a basis for further investigation into the clinicopathological factors that influence their long-term outcomes.

Notably, preoperative serum PSA levels showed no significant difference between RCP patients with and without IPCa. This finding is consistent with prior reports and underscores the limited predictive value of routine PSA screening for detecting IPCa in the context of bladder cancer (13, 14). Furthermore, analysis of preoperative bladder imaging in 69 patients with bladder cancer complicated by IPCa revealed no cases radiologically suggestive of concurrent prostate malignancy, highlighting the low sensitivity of conventional imaging modalities for identifying coexisting IPCa. This limitation of conventional imaging extends to more specialized techniques: among the subset of patients who underwent preoperative prostate MRI, all were assigned a PI-RADS score of 1 or 2, indicating a low level of clinical suspicion for prostate cancer, which further demonstrates the diagnostic challenge posed by IPCa in this patient population. Collectively, these observations align with existing literature indicating that no reliable preoperative biomarkers currently exist to definitively rule out IPCa. To improve detection, optimized pathological sampling protocols during specimen processing should also be considered to enhance IPCa identification rates.

Radical surgery remains the primary treatment for bladder urothelial carcinoma, yet its efficacy is often limited by high recurrence rates. Advances in targeted therapy have highlighted HER2 as a potential biomarker, given its widespread tissue expression (15, 16). A meta-analysis by Zhao et al. involving 2,242 patients with muscle-invasive urothelial carcinoma reported a HER2 protein overexpression rate of 41.2%, underscoring its potential prognostic and predictive value (17). Luigi Cormio et al. (18) demonstrated a statistically significant positive correlation between HER2 overexpression and both tumor recurrence and disease progression in bladder urothelial carcinoma cases.

In this study, we evaluated HER2 protein expression in 237 patients with high-grade urothelial carcinoma using a four-point scoring system and categorized them into three groups: 0/1+, 2+, and 3 +. Initial analysis revealed significant associations between HER2 expression patterns and specific clinicopathological features within this uniformly high-grade cohort. Notably, a nonlinear relationship was observed between HER2 expression level and tumor size, with the HER2 2+ subgroup exhibiting a significantly smaller median maximum tumor diameter compared to both the 0/1+ and 3+ subgroups. Additionally, conventional urothelial carcinoma was predominant in the HER2 0/1+ subgroup, suggesting that low HER2 expression may correlate with classic histological morphology. These findings indicate that even among high-grade tumors, HER2 expression demonstrates considerable heterogeneity and may offer additional stratification value beyond tumor grade alone.

To further assess the robustness of these observations, we conducted two supplementary analyses. First, a sensitivity analysis applying the more stringent clinical criterion of “IHC 3+ only as positive” showed that the associations described above did not retain statistical significance(all *P*>0.05). Second, detailed subgroup analyses based on bladder cancer type (NMIBC vs. MIBC) and prostate cancer risk groups revealed no statistically significant differences in HER2 expression across these subpopulations(all *P*>0.05). Collectively, these supplementary results suggest that the identified associations between HER2 expression patterns and pathological features are sensitive to the chosen immunohistochemical scoring threshold and may not manifest consistently across all patient subgroups.

Overall, this study clarifies the complex role of HER2 in high-grade urothelial carcinoma by demonstrating heterogeneous expression patterns linked to distinct pathological characteristics. Rather than merely confirming the known association between HER2 amplification and high tumor grade (19–21), our findings propose that HER2 status could contribute to finer biological stratification within high-grade disease. This evidence reinforces the clinical relevance of HER2 in urothelial carcinoma, with expression patterns potentially informing more nuanced risk assessment. However, the sensitivity of these associations to scoring criteria, along with the limitation of lacking *in situ* hybridization confirmation for equivocal (2+) cases, highlights the need for standardized HER2 assessment protocols and further validation in larger, multi-center cohorts to solidify its prognostic and predictive utility.

Our investigation aligns with a broader shift in bladder cancer research from purely morphological assessment toward an integrated, multidimensional understanding of tumor biology. Recent studies emphasize the importance of combining morphological and molecular phenotypic data in evaluating surgical specimens. For example, Sanguedolce et al. (22) demonstrated that molecular subtypes defined by immunohistochemical markers (CK5/6, GATA3, CK20) correlated significantly with patterns of muscularis propria invasion, directly linking molecular phenotypes to prognostically relevant morphological features. This evidence supports an evolving paradigm in bladder cancer pathology: moving beyond TNM staging alone toward a “clinical-pathological-molecular” integrated framework that incorporates both detailed morphological parameters and molecular subtype information.

In summary, RCP remains the standard surgical approach for bladder cancer. Given the unique pathological context of this dual-cancer cohort, the suitability of PSC and its potential implications for IPCa residual or recurrence warrants careful evaluation, as the present study lacks comparative data on different surgical techniques. Therefore, more comprehensive examinations should be performed for such patients to reduce the corresponding risks. Additionally, the value of HER2 expression as a molecular biomarker in the diagnosis and management of urothelial carcinoma warrants further exploration.

## 5 Conclusions

In this study, we found that IPCa in patients who have undergone RCP is not uncommon (29.1%). Age was a risk factor for bladder cancer with IPCa. It is of great significance to evaluate the risk factors for the selection of surgical methods and follow-up management. Expression pattern of HER2 correlates with key tumor characteristics, which may provide valuable insights for risk stratification and personalized treatment decision-making in patients with this malignancy. And these survival data indicated that patients with IPCa had a relatively favorable overall prognosis, laying a foundation for further exploration of the clinicopathological factors influencing their long-term survival.

## Data Availability

The raw data supporting the conclusions of this article will be made available by the authors, without undue reservation.
