# Correction: Clinicopathological characteristics and assessment of risk factors for incidentally discovered prostatic cancer in patients undergoing radical cystectomy with urothelial carcinoma of the bladder

**DOI:** 10.3389/fonc.2026.1937745

**Published:** 2026-08-21

**Authors:** Ying Qiu, Nai-Chun Zhang, Xiao Tan, Ning Jin, Da Chen, Jing Zhao

**Affiliations:** 1 Department of Pathology, Linyi People′s Hospital, Linyi, Shandong, China; 2 Department of Infectious Disease, Linyi People’s Hospital, Linyi, Shandong, China

**Keywords:** assessment of risk factors, bladder urothelial carcinoma, HER2, incidental prostatic adenocarcinoma, radical cystectomy

Author “Ying Qiu” was erroneously assigned as corresponding author. The correct corresponding author is “Nai-Chun Zhang”.

The **Reference** for 1 was erroneously written as “Hermans A, Heisterkamp N, von Linden M, van Baal S, Meijer D, van der Plas D, et al. Unique fusion of bcr and c-abl genes in Philadelphia chromosome positive acute lymphoblastic leukemia. Cell. (1987) 51:33–40. doi:10.1016/0092-8674(87)90007-9”.

It should be “Porreca A, Palmer K, Artibani W, Antonelli A, Bianchi L, Brunocilla E, et al. Protocol of the italian radical cystectomy registry (RIC): a non-randomized, 24-month, multicenter study comparing robotic-assisted, laparoscopic, and open surgery for radical cystectomy in bladder cancer. BMC Cancer. (2021) 21: 51. doi: 10.1186/s12885-020-07748-7”.

The **Reference** for 2 was erroneously written as “Short NJ, Kantarjian H, Jabbour E. Optimizing the treatment of acute lymphoblastic leukemia in younger and older adults: new drugs and evolving paradigms. Leukemia. (2021) 35:3044–58. doi: 10.1038/s41375-021-01277-3”.

It should be “Abdelaziz AY, Shaker H, Seifelnasr M, Elfol H, Nazim M, Mahmoued M. Early oncological and functional outcomes of prostate capsule sparing cystectomy compared with standard radical cystectomy. Curr Urol. (2019) 13: 37-45. doi: 10.1159/000499296”.

The **Reference** for 3 was erroneously written as “Jabbour E, Pui CH, Kantarjian H. Progress and innovations in the management of adult acute lymphoblastic leukemia. JAMA Oncol. (2018) 4:1413–20. doi: 10.1001/jamaoncol.2018.1915”.

It should be “Quesada-Olarte J, Alvarez-Maestro M, Gomez-Rivas J, Toribio-Vazquez C, Aguilera BA, Martinez-Pineiro L. Organ-sparing cystectomy techniques: functional and oncological outcomes, review and current recommendations. Arch Esp Urol. (2020) 73: 961-70”.

The **Reference** for 4 was erroneously written as “Fielding AK, Rowe JM, Buck G, Foroni L, Gerrard G, Litzow MR, et al. UKALLXII/ECOG2993: addition of imatinib to a standard treatment regimen enhances long-term outcomes in Philadelphia positive acute lymphoblastic leukemia. Blood. (2014) 123:843–50. doi: 10.1182/blood-2013-09-529008”.

It should be “Hoshi S, Bilim V, Hoshi K, Ogawa Y, Kato T, Urano K, et al. Double primary cancer of the prostate and urothelial cancer: a single institution experience. J Pers Med. (2024) 14. doi: 10.3390/jpm14050510”.

The **Reference** for 5 was erroneously written as “Ribera JM, Oriol A, González M, Vidriales B, Brunet S, Esteve J, et al. Concurrent intensive chemotherapy and imatinib before and after stem cell transplantation in newly diagnosed Philadelphia chromosome-positive acute lymphoblastic leukemia. Final results of the CSTIBES02 trial. Haematologica. (2010) 95:87–95. doi: 10.3324/haematol.2009.011221”.

It should be “Mishra P, Jiongming L, Jianhe L, Kewei F, Yongming J, Wang G, et al. Prostate cancer among patients undergoing radical cystoprostatectomy for bladder cancer in the department of urology in a tertiary care centre. JNMA J Nepal Med Assoc. (2023) 61: 782-6. doi: 10.31729/jnma.8309”.

The **Reference** for 6 was erroneously written as “Lim SN, Joo YD, Lee KH, Kim DY, Lee JH, Lee JH, et al. Long-term follow-up of imatinib plus combination chemotherapy in patients with newly diagnosed Philadelphia chromosome-positive acute lymphoblastic leukemia. Am J Hematol. (2015) 90:1013–20. doi: 10.1002/ajh.24137”.

It should be “Bruins HM, Djaladat H, Ahmadi H, Sherrod A, Cai J, Miranda G, et al. Incidental prostate cancer in patients with bladder urothelial carcinoma: comprehensive analysis of 1,476 radical cystoprostatectomy specimens. J Urol. (2013) 190: 1704-9. doi: 10.1016/j.juro.2013.05.034”.

The **Reference** for 7 was erroneously written as “Daver N, Thomas D, Ravandi F, Cortes J, Garris R, Jabbour E, et al. Final report of a phase II study of imatinib mesylate with hyper-CVAD for the front-line treatment of adult patients with Philadelphia chromosome-positive acute lymphoblastic leukemia. Haematologica. (2015) 100:653–61. doi: 10.3324/haematol.2014.118588”.

It should be “Moschini M, Shariat SF, Freschi M, Soria F, Abufaraj M, Gandaglia G, et al. Impact of prostate involvement on outcomes in patients treated with radical cystoprostatectomy for bladder cancer. Urol Int. (2017) 98: 290-7. doi: 10.1159/000454736”.

The **Reference** for 8 was erroneously written as “Bassan R, Rossi G, Pogliani EM, Di Bona E, Angelucci E, Cavattoni I, et al. Chemotherapy-phased imatinib pulses improve long-term outcome of adult patients with Philadelphia chromosome-positive acute lymphoblastic leukemia: Northern Italy Leukemia Group protocol 09/00. J Clin Oncol. (2010) 28:3644–52. doi: 10.1200/JCO.2010.28.1287”.

It should be “Zhou XD, Wang JJ, Xu XQ, Liu N, Ma WL. [Incidental prostate cancer: a clinicopathological study]. Zhonghua Nan Ke Xue. (2021) 27: 145-9”.

The **Reference** for 9 was erroneously written as “Ravandi F, Othus M, O’Brien SM, Forman SJ, Ha CS, Wong JYC, et al. US intergroup study of chemotherapy plus dasatinib and allogeneic stem cell transplant in Philadelphia chromosome positive ALL. Blood Adv. (2016) 1:250 -9.doi:10.1182/bloodadvances.2016001495”.

It should be “Packiam VT, Tsivian M, Avulova S, Sharma V, Tarrell R, Cheville JC, et al. Long-term outcomes of incidental prostate cancer at radical cystectomy. Urol Oncol. (2020) 38: 817-48. doi: 10.1016/j.urolonc.2020.05.018”.

The **Reference** for 10 was erroneously written as “Porkka K, Koskenvesa P, Lundán T, Rimpiläinen J, Mustjoki S, Smykla R, et al.Dasatinib crosses the blood-brain barrier and is an efficient therapy for central nervous system Philadelphia chromosome-positive leukemia. Blood. (2008) 112:1005–12.doi: 10.1182/blood-2008-02-140665”.

It should be “Jonck S, Helgstrand JT, Roder MA, Klemann N, Gronkaer TB, Brasso K. The prognostic impact of incidental prostate cancer following radical cystoprostatectomy: a nationwide analysis. Scand J Urol. (2018) 52: 358-63. doi: 10.1080/21681805.2018.1534885”.

The **Reference** for 11 was erroneously written as “Shen S, Chen X, Cai J, Yu J, Gao J, Hu S, et al. Effect of dasatinib vs imatinib in the treatment of pediatric philadelphia chromosome-positive acute lymphoblastic leukemia: A randomized clinical trial. JAMA Oncol. (2020) 6:358–66. doi:10.1001/jamaoncol.2019.5868”.

It should be “Kaelberer JB, O'Donnell MA, Mitchell DL, Snow AN, Mott SL, Buatti JM, et al. Incidental prostate cancer diagnosed at radical cystoprostatectomy for bladder cancer: disease-specific outcomes and survival. Prostate Int. (2016) 4: 107-12.doi:10.1016/j.prnil.2016.06.002”.

The **Reference** for 12 was erroneously written as “Benjamini O, Dumlao TL, Kantarjian H, O’Brien S, Garcia-Manero G, Faderl S,et al. Phase II trial of hyper CVAD and dasatinib in patients with relapsed Philadelphia chromosome positive acute lymphoblastic leukemia or blast phase chronic myeloid leukemia. Am J Hematol. (2014)89:282–7. doi: 10.1002/ajh.23624”.

It should be “Jing Y, Zhang R, Ma P, Cai MC, Zhuang G, Chen H. Prevalence and clonality of synchronous primary carcinomas in the bladder and prostate. J Pathol. (2018) 244: 5-10. doi: 10.1002/path.4997”.

The **Reference** for 13 was erroneously written as “Talpaz M, Shah NP, Kantarjian H, Donato N, Nicoll J, Paquette R, et al. Dasatinib in imatinib-resistant Philadelphia chromosome-positive leukemias. N Engl J Med. (2006) 354:2531–41. doi: 10.1056/NEJMoa055229”.

It should be “Capitanio U, Ventimiglia E, Montorsi F. The significance of a high preoperative PSA level for the detection of incidental prostate cancer in LUTS patients with large prostates. World J Urol. (2022) 40(4):1063-4. doi: 10.1007/s00345-021-03606-8”.

The **Reference** for 14 was erroneously written as “Soverini S, Colarossi S, Gnani A, Castagnetti F, Rosti G, Bosi C, et al. Resistance to dasatinib in Philadelphia-positive leukemia patients and the presence or the selection of mutations at residues 315 and 317 in the BCR-ABL kinase domain. Haematologica. (2007) 92:401–4. doi: 10.3324/haematol.10822”.

It should be “Ni R, Lai S, Hu H, Seery S, Xu T, Hu H. Clinicopathologic features and prognosis of incidental prostate cancer after radical cysto-prostatectomy: a comparative study between china and the west. Transl Androl Urol. (2024) 13: 2694-704. doi: 10.21037/tau-24-441”.

The **Reference** for 15 was erroneously written as “Lilly MB, Ottmann OG, Shah NP, Larson RA, Reiffers JJ, Ehninger G, et al. Dasatinib 140 mg once daily versus 70 mg twice daily in patients with Ph-positive acute lymphoblastic leukemia who failed imatinib: Results from a phase 3 study. Am J Hematol. (2010) 85:164–70. doi: 10.1002/ajh.21615”.

It should be “Zhu K, Yang X, Tai H, Zhong X, Luo T, Zheng H. HER2-targeted therapies in cancer: a systematic review. Biomark Res. (2024) 12: 16. doi: 10.1186/s40364-024-00565-1”.

The **Reference** for 16 was erroneously written as “Hughes TP, Laneuville P, Rousselot P, Snyder DS, Rea D, Shah NP, et al. Incidence, outcomes, and risk factors of pleural effusion in patients receiving dasatinib therapy for Philadelphia chromosome-positive leukemia. Haematologica. (2019) 104:93–101.doi: 10.3324/haematol.2018.188987”.

It should be “Gan K, Gao Y, Liu K, Xu B, Qin W. The clinical significance and prognostic value of HER2 expression in bladder cancer: a meta-analysis and a bioinformatic analysis. Front Oncol. (2021) 11: 653491. doi: 10.3389/fonc.2021.653491”.

The **Reference** for 17 was erroneously written as “Kantarjian H, Short NJ, Jain N, Sasaki K, Huang X, Haddad FG, et al. Frontline combination of ponatinib and hyper-CVAD in Philadelphia chromosome-positive acute lymphoblastic leukemia: 80-months follow-up results. Am J Hematol. (2023) 98:493–501. doi: 10.1002/ajh.26816”.

It should be “Zhao J, Xu W, Zhang Z, Song R, Zeng S, Sun Y, et al. Prognostic role of HER2 expression in bladder cancer: a systematic review and meta-analysis. Int Urol Nephrol. (2015) 47: 87-94. doi: 10.1007/s11255-014-0866-z”.

The **Reference** for 18 was erroneously written as “Ribera JM, Garcí a-Calduch O, Ribera J, Montesinos P, Cano-Ferri I, Martı nez P,et al. Ponatinib, chemotherapy, and transplant in adults with Philadelphia chromo-some-positive acute lymphoblastic leukemia. Blood Adv. (2022) 6:5395-402.doi:10.1182/bloodadvances.2022007764”.

It should be “Cormio L, Sanguedolce F, Cormio A, Massenio P, Bufo P. Human epidermal growth factor receptor 2 expression is more important than bacillus calmette guerin treatment in predicting the outcome of t1g3 bladder cancer. Oncotarget. (2017) 8:25433-41. doi: 10.18632/oncotarget.15989”.

The **Reference** for 19 was erroneously written as “Sasaki K, Kantarjian HM, Short NJ, Samra B, Khoury JD, Kanagal Shamanna R, et al.Prognostic factors for progression in patients with Philadelphia chromosome-positive acute lymphoblastic leukemia in complete molecular response within 3 months of therapy with tyrosine kinase inhibitors. Cancer. (2021) 127:2648–56. doi: 10.1002/cncr.33529”.

It should be “Sanguedolce F, Zanelli M, Palicelli A, Bisagni A, Zizzo M, Ascani S, et al. HER2 expression in bladder cancer: a focused view on its diagnostic, prognostic, and predictive role. Int J Mol Sci. (2023) 24. doi: 10.3390/ijms24043720”.

The **Reference** for 20 was erroneously written as “Ribera JM, Prawitz T, Freitag A, Sharma A, Dobi B, Rizzo F, et al. Ponatinib vs.Imatinib as frontline treatment for philadelphia chromosome-positive acute lympho-blastic leukemia: A matching adjusted indirect comparison. Adv Ther. (2023) 40:3087–103. doi: 10.1007/s12325-023-02497-y”.

It should be “Zinnall U, Weyerer V, Comperat E, Camparo P, Gaisa NT, Knuechel-Clarke R, et al. Micropapillary urothelial carcinoma: evaluation of HER2 status and immunohistochemical characterization of the molecular subtype. Hum Pathol. (2018) 80: 55-64. doi: 10.1016/j.humpath.2018.05.022”.

The **Reference** for 21 was erroneously written as “Sasaki K, Jabbour EJ, Ravandi F, Short NJ, Thomas DA, Garcia-Manero G, et al.Hyper-CVAD plus ponatinib versus hyper-CVAD plus dasatinib as frontline therapy for patients with Philadelphia chromosome-positive acute lymphoblastic leukemia: A propensity score analysis. Cancer. (2016) 122:3650–6. doi: 10.1002/cncr.30231”.

It should be “Amer IS, Helmy ET, Abdel AA, Shibel P. Immunohistochemical evaluation of perlecan (heparan sulphate proteoglycan 2) expression in invasive female breast carcinoma. Asian Pac J Cancer Prev. (2023) 24: 4277-83. doi: 10.31557/APJCP.2023.24.12.4277”.

The **Reference** for 22 was erroneously written as “Chalandon Y, Thomas X, Hayette S, Cayuela JM, Abbal C, Huguet F, et al. Randomized study of reduced-intensity chemotherapy combined with imatinib in adults with Ph-positive acute lymphoblastic leukemia. Blood. (2015) 125:3711–9. doi: 10.1182/blood-2015-02-627935”.

It should be “Sanguedolce F, Falagario U, Zanelli M, Palicelli A, Zizzo M, Ascani S, et al. Clinicopathological features and survival analysis in molecular subtypes of muscle-invasive bladder cancer. Int J Mol Sci. (2023) 24:6610. doi: 10.3390/ijms24076610”.

The original version of this article has been updated.

